# Age-Related Differences in Oral Microbiota Among Obese Patients with Periodontitis: A Systematic Review

**DOI:** 10.3390/nu18081256

**Published:** 2026-04-16

**Authors:** Felicia Gabriela Beresescu, Razvan Marius Ion, Adriana Monea, Alina Ormenisan, Despina Luciana Bereczki-Temistocle, Liana Beresescu, Andrea Bors

**Affiliations:** 1 Faculty of Dental Medicine, George Emil Palade University of Medicine, Pharmacy, Science, and Technology of Targu Mureș, 540142 Targu Mureș, Romania; felicia.beresescu@umfst.ro (F.G.B.); adriana.monea@umfst.ro (A.M.); alina.ormenisan@umfst.ro (A.O.); despina.bereczki-temistocle@umfst.ro (D.L.B.-T.); andrea.bors@umfst.ro (A.B.); 2 Second Department of Surgery, George Emil Palade University of Medicine, Pharmacy, Science and Technology of Targu Mureș, 540142 Targu Mureș, Romania; 3 Doctoral School of Medicine and Pharmacy, George Emil Palade University of Medicine, Pharmacy, Sciences and Technology of Targu Mureș, 540142 Targu Mureș, Romania; 4 2nd Department of Surgery, Mureș County Emergency Hospital, 540136 Targu Mureș, Romania

**Keywords:** aging, obesity, periodontitis, oral microbiota, dysbiosis, systemic inflammation

## Abstract

**Background:** Obesity and periodontitis are linked through inflammatory and metabolic pathways, and the oral microbiota may mediate this interaction. Age-related changes in immunity, salivary function, and cumulative exposure may modify obesity-associated periodontal dysbiosis. **Objective:** We sought to synthesize the potential for age-related differences in the oral microbiota of adult obese patients with periodontitis and assess the strength of current literature in supporting age-specific interpretations. **Methods:** A systematic search of PubMed/MEDLINE, Scopus, and Embase identified 1088 records. After screening and full-text assessment, 50 studies that met the criteria for focused qualitative synthesis remained. Within that review corpus, 10 representative adult human studies provided the most direct evidence linking obesity or overweight, periodontal phenotype, oral microbiota, and age-relevant interpretation. Risk of bias was appraised with the Newcastle–Ottawa Scale. **Results:** Direct head-to-head microbiome comparisons between younger and older obese adults with periodontitis are rare. Direct evidence links obesity to greater periodontal inflammatory burden, enrichment of classical periopathogens and bridging taxa, and shifts in community structure. Contextual aging studies have suggested that older adults may more often harbor lower-diversity, persistence-oriented communities enriched in stress-tolerant, proteolytic, or opportunistic taxa, whereas younger obese adults more often show inflammation-amplifying consortia enriched in classical periopathogens and bridging taxa. However, these patterns remain largely hypothesis-generating because the evidence base is heterogeneous and predominantly cross-sectional. **Conclusions:** Age likely modifies the obesity–periodontitis–microbiota axis, but direct comparative evidence on adults remains limited. The current literature supports cautious age-aware interpretation within a systematic review framework rather than definitive age-specific microbial signatures or treatment algorithms.

## 1. Introduction

Age, obesity, and periodontitis are independently associated with shifts in the oral microbiota; however, their combined effects and interactions remain incompletely understood. Several reviews and clinical studies have highlighted that aging is linked to an increased prevalence and severity of periodontitis, potentially due to changes in the subgingival microbiota, with some taxa (e.g., *Porphyromonas gingivalis*) increasing and others (e.g., *Aggregatibacter actinomycetemcomitans*) decreasing with age [1,2,3]. The World Health Organization (WHO) estimates that more than 1.9 billion adults are overweight and over 650 million live with obesity (~13% of the adult population) [1]. Periodontitis affects up to ~50% of adults, with severe disease (stage III/IV) affecting ~11% of the global population [2,3]. The interaction between these conditions varies across one’s lifespan. Age-related shifts in immunity, salivary function, and cumulative exposure can alter the oral microbial ecology, thereby influencing the link between obesity and periodontal breakdown.

Obesity is increasingly conceptualized as ‘metaflammation,’ a state of chronic, low-grade systemic inflammation driven by adipose tissue dysfunction and altered immunometabolic signaling [4,5,6]. Adipose tissue acts as an endocrine organ, releasing pro-inflammatory cytokines and adipokines (e.g., TNF-α, IL-6, leptin, and resistin), which contribute to oxidative stress, endothelial dysfunction, and immune dysregulation [7,8]. This systemic milieu can prime periodontal tissues for exaggerated inflammatory responses and may facilitate dysbiosis by altering local nutrient availability and host defense [9,10].

Periodontitis is a multifactorial inflammatory disease in which dysbiotic biofilms interact with a susceptible host response, leading to connective tissue breakdown and alveolar bone loss [11]. In healthy individuals, a commensal-dominated oral microbiota supports colonization resistance and immune homeostasis. In diseased conditions, dysbiosis, a shift in microbial composition and function away from homeostasis, is characterized by the enrichment of anaerobic, proteolytic taxa and increased inflammatory potential [12,13,14]. Late-colonizing periodontopathogens (e.g., *Porphyromonas gingivalis*, *Tannerella forsythia*, and *Treponema denticola*) and synergistic bridging organisms contribute to biofilm maturation, immune subversion, and sustained inflammation [15,16].

Evidence supports the notion that there is a bidirectional relationship between obesity and periodontitis. Obesity increases susceptibility to periodontitis through immune and metabolic dysregulation, whereas periodontal inflammation may exacerbate systemic inflammation and insulin resistance, thereby worsening cardiometabolic risk [17,18,19]. The oral microbiota is central to this axis, and obesity-associated changes in inflammatory tone, saliva, and behavior can reshape microbial communities, while dysbiotic biofilms can reinforce host inflammation through reciprocal feedback.

Crucially, age dynamically modulates the obesity–periodontitis–microbiota axis. It profoundly influences both immune competence and microbial ecology. Aging is associated with immunosenescence and inflammaging, reduced mucosal defense capacity, salivary gland hypofunction (often due to polypharmacy), and increased multimorbidity. These factors collectively influence biofilm composition, resilience, and treatment responses [20,21,22]. Beyond chronological age, geriatric phenotypes, such as frailty, and other biological age markers may better capture host vulnerability to persistent dysbiosis [23,24].

These age-related microbial patterns have important practical implications. Younger patients may present with more acute clinical inflammation and may benefit from early and intensive control of dysbiotic biofilms alongside metabolic risk management. Older adults may exhibit less bleeding despite significant bone loss and are more likely to experience xerostomia, impaired oral hygiene capacity, and polymicrobial opportunism, complicating diagnosis and long-term maintenance [25,26].

Despite growing interest in the obesity–periodontitis axis, direct microbiome studies explicitly comparing younger and older obese adults with periodontitis remain scarce. Most studies evaluate obesity, periodontal phenotype, or aging separately, making age-stratified interpretation indirect [27,28].

Aging is treated as both time-dependent exposure (chronological age) and a set of biological modifiers (e.g., frailty, multimorbidity, polypharmacy, and salivary hypofunction) that shape the host immune tone and oral ecosystem. Obesity may promote periodontal breakdown through systemic immune dysregulation and an oral environment amenable to dysbiosis [29]. Conversely, periodontitis may contribute to metabolic deterioration through inflammatory spillovers. Over the course of one’s life, the oral microbiota acts as a mechanistic bridge between these conditions by modulating biofilm virulence and host responses [4,9,27,29].

**Figure 1:**
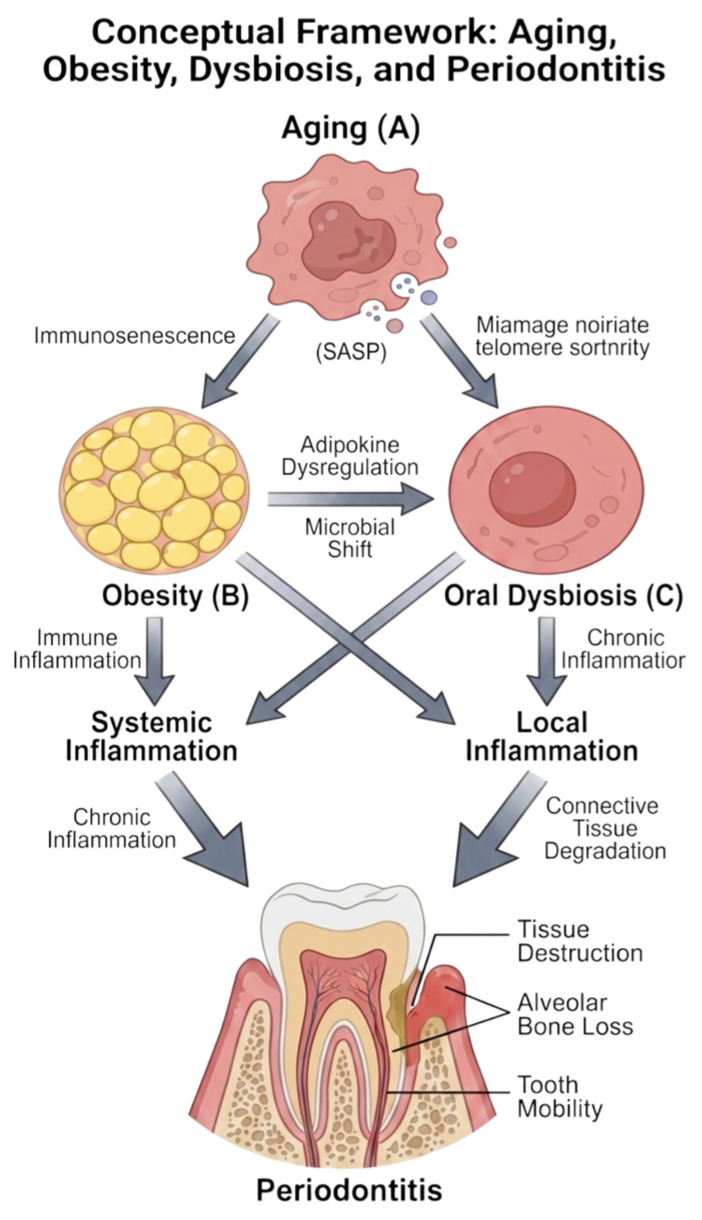
Conceptual framework: Aging, Obesity, Dysbiosis, and Periodontitis. created in BioRender. Beresescu, F.G. 2026. (https://BioRender.com/i1b24rq accessed on 11 April 2026) is licensed under CC BY 4.0.

Figure 1 illustrates our conceptual framework, highlighting aging as both chronological time and a set of biological modifiers, including frailty, multimorbidity, polypharmacy, tooth retention, and salivary dysfunction, that may reshape host responses and microbial ecology.

This systematic review summarizes the available evidence on age-related differences in the oral microbiota of adult obese patients with periodontitis while explicitly distinguishing direct evidence from contextual adult evidence used for age-aware interpretation.

## 2. Materials and Methods

### 2.1. Literature Search Strategy

**Figure 2:**
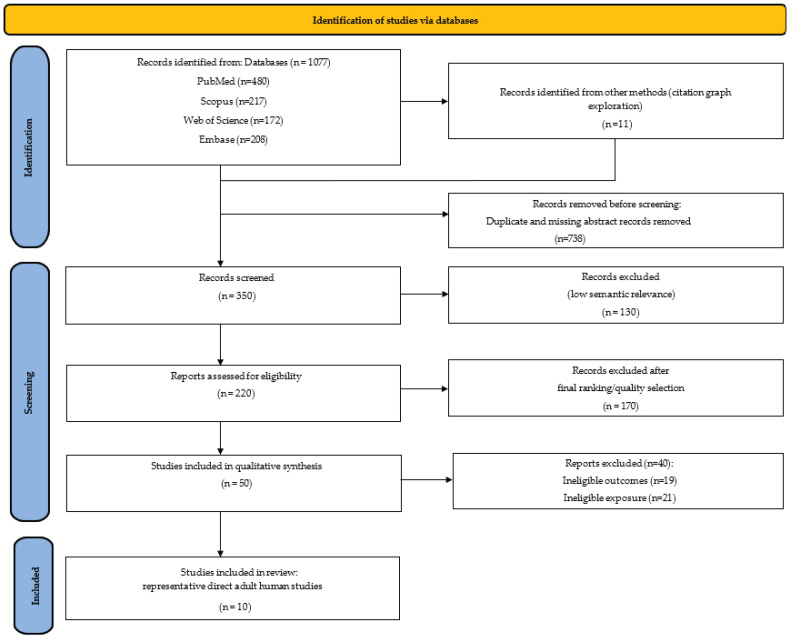
PRISMA flowchart.

We used a structured, systematic search to improve transparency and reproducibility while focusing on qualitative synthesis rather than meta-analysis. We searched PubMed/MEDLINE, Scopus, and Embase using combinations of terms related to obesity, periodontitis, oral microbiota, aging, and dysbiosis. The search identified 1088 records. After removing duplicates and screening titles and abstracts, we assessed 220 full texts for eligibility. Fifty adult studies met the pre-defined relevance criteria and were retained for qualitative synthesis within this systematic review. Of these, 10 representative adult human studies were highlighted in Table 1 because they most directly combined obesity or overweight, periodontal phenotype, and oral microbiota data with age-relevant reporting (Supplementary Table S1). The full evidence map of the 50 studies retained for qualitative synthesis is provided in Supplementary Table S2. The study-selection process is summarized in the PRISMA flowchart (Figure 2).

### 2.2. Eligibility Criteria

Eligible studies were human studies involving adults aged 18 years or older who reported oral microbiota findings in relation to obesity or overweight and periodontitis, with age distributions, age-relevant subgroup information, or variables informative for age-related interpretation. Pediatric-only studies were excluded from the core synthesis. Contextual aging studies in adults were used only to interpret age-related changes when direct obesity–periodontitis microbiome comparisons were unavailable.

### 2.3. Study Selection and Data Extraction

Two reviewers independently screened records and assessed full texts, resolving disagreements by consensus. The variables extracted included study design, sample characteristics, age distribution, obesity definition, periodontal phenotype, sampling site, microbiome method, major taxa reported, and adjustment for confounding. Ten adult human studies provided the most direct age-relevant evidence, which is highlighted in Table 1.

### 2.4. Data Synthesis and Age Stratification

As directly age-stratified cohorts of obese patients with periodontitis are uncommon, we distinguished direct evidence from contextual age studies on adults. Working-age bands (under 45 years and over 65 years) were used only as an interpretive framework. Biological age modifiers such as frailty, multimorbidity, polypharmacy, edentulism, and salivary dysfunction were recorded where available, but they were not consistently reported across studies.

### 2.5. Quality Assessment

Although some quantitative data (e.g., relative abundances or effect sizes for specific taxa) were reported, the variability in measurement and reporting standards, combined with predominantly cross-sectional designs and moderate to high risk of bias, limited the feasibility and validity of meta-analytic approaches. Accordingly, this systematic review used a structured qualitative synthesis to integrate findings meaningfully while highlighting the need for harmonized methodologies in future research.

Risk of bias was evaluated using the Newcastle–Ottawa Scale (NOS) for cohort and case–control studies and an NOS-informed appraisal for cross-sectional studies. The appraisal focused on selection, comparability, and outcome/exposure assessment domains. A study-level summary is provided in Supplementary Table S1. It is used here to inform interpretations rather than exclude studies mechanically.

## 3. Results

The included evidence base was heterogeneous and dominated by cross-sectional adult studies, supplemented by contextual aging research. Representativeness was often limited by clinic-based or convenience sampling, and adjustments for smoking, diabetes, oral hygiene, medication burden, and socioeconomic factors were inconsistent. These issues call for cautious interpretation of any age-aware microbial patterns.

Throughout the focused literature, obesity is generally associated with periodontal inflammatory burden and shifts toward dysbiotic oral communities. However, direct head-to-head comparisons between younger and older obese adults with periodontitis are rare, so age-aware conclusions rely on a combination of direct adult evidence and contextual aging studies [30,31,32,33,34].

### 3.1. Oral Microbiota in Health and Disease: Foundations for Understanding Dysbiosis with Regard to Obesity and Aging

The oral cavity contains a complex microbial ecosystem distributed across saliva, mucosal surfaces, the tongue dorsum, and subgingival niches [12,13,15]. In eubiosis, these communities help maintain colonization resistance and mucosal immune homeostasis. In periodontitis, ecological pressure shifts toward inflammation-adapted, proteolytic communities that exploit host-derived nutrients and sustain tissue-destructive inflammation [10,11,12,13,14,15].

Canonical periodontopathogens such as *P. gingivalis*, *T. forsythia*, and *Treponema denticola*, together with bridging and synergistic taxa, are central to this dysbiotic transition [10,11,14,15]. Other organisms associated with persistence or deep-pocket disease, including *Filifactor alocis*, *Dialister* spp., and *Eubacterium nodatum*, are increasingly implicated in chronic periodontal disruption.

Obesity can intensify this ecological shift through chronic low-grade inflammation, altered adipokine signaling, oxidative stress, and salivary changes that may favor pathogen persistence. Several adult studies also report obesity-associated enrichment of inflammation-linked anaerobes and a higher periodontal inflammatory burden [22,25,26,27,28].

Aging adds further complexity through immunosenescence, inflammaging, multimorbidity, polypharmacy, tooth loss, and salivary dysfunction. These factors can modify microbial selection pressures and clinical expression even when direct age-stratified obesity–periodontitis microbiome studies are unavailable [17,18,19,20,21,24,29,30].

Thus, the available literature supports the notion of a biologically plausible effect of age on obesity-associated periodontal dysbiosis, but it does not yet establish definitive age-specific microbial signatures.

### 3.2. Age-Related Oral Microbial Profiles in Obesity-Associated Periodontitis

Age is a major modifier of periodontal dysbiosis, influencing community composition and function. In obesity, additional metabolic and immunological pressures (metaflammation, oxidative stress, and altered salivary substrates) may further destabilize the subgingival ecosystem [20,22]. Direct adult microbiome studies jointly evaluating obesity, periodontitis, and age are limited. Given the scarcity of direct age-stratified studies in obese patients with periodontitis, our interpretation largely relies on synthesizing indirect evidence from contextual aging studies. Therefore, the most defensible interpretation is a hypothesis-generating working model rather than a definitive typology: among younger adults, obesity may amplify pathogen-rich, inflammation-active dysbiosis, whereas in older adults, cumulative exposure and host vulnerability may favor persistence-oriented communities. Readers are advised to take this distinction into consideration as they appraise the proposed patterns.

This interpretation is conditioned by substantial methodological heterogeneity, including variability in study design, obesity definitions (e.g., body mass index cut-offs, body composition), periodontal phenotyping (e.g., case definitions, severity staging), sampling sites (e.g., saliva, subgingival plaque), sequencing platforms (e.g., 16S rRNA gene regions, whole-genome sequencing), and inconsistent confounder adjustment. Analysis of the included primary studies revealed moderate to high risk of bias, particularly regarding selection (convenience sampling, lack of clear inclusion/exclusion criteria for age subgroups) and comparability (inconsistent adjustment for key confounders like smoking, diabetes, or socioeconomic status). These biases necessitate cautious interpretation of reported associations, as detailed in Supplementary Table S1.

In younger obese adults with periodontitis, direct studies more often report enrichment of classical periopathogens and bridging taxa, including *P. gingivalis*, *T. forsythia*, *P. intermedia*, *Campylobacter*, and related inflammation-amplifying consortia [22,25,26,27,28]. These communities fit a higher-activity model in which metaflammation, oxidative stress, and altered salivary substrates may intensify biofilm maturation and overt clinical inflammation.

Regarding older adults, the literature more often provides contextual rather than direct evidence. Adult aging studies suggest lower-diversity, proteolytic, and stress-tolerant communities have a greater influence, with recurrent signals involving *F. alocis*, *Dialister* spp., *E. nodatum*, and opportunistic colonizers in vulnerable hosts [24,29,30,31].

Table 1 distinguishes representative direct studies from contextual adult studies used to interpret age-related modification. This distinction is important because the current literature does not justify treating the proposed younger and older patterns as established microbial signatures.

Older-adult patterns are also shaped by edentulism, treatment history, multimorbidity, medication burden, and reduced salivary reserve, all of which can alter oral ecology independently of obesity [32,33].

Accordingly, age-aware interpretations should be viewed as hypothesis-generating and biologically plausible, not definitive.

**Figure 3:**
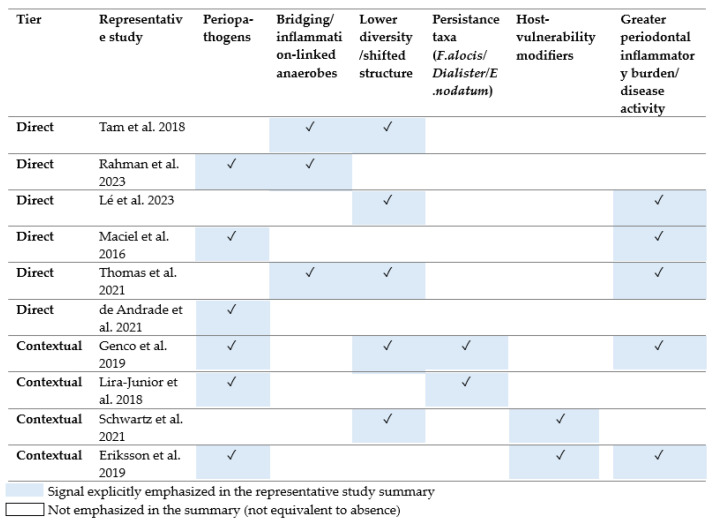
Recurrent microbiota-related signals across representative studies [22,23,24,25,26,27,28,29,30,31].

Across representative studies, recurrent signals cluster into a limited set of themes rather than stable age-specific microbial signatures. In direct obesity-related cohorts, repeated signals include classical periopathogens, bridging taxa, lowered diversity or community shift, and greater inflammatory burden. In contextual adult studies, recurrent signals more often involve persistence-associated taxa, edentulism or tooth-loss context, and host-vulnerability modifiers. These convergence points are summarized in Figure 3.

In older adults, a persistent trajectory characterized by low-diversity communities enriched in *F. alocis*, *Peptostreptococcus*, *Eubacterium*, and opportunistic organisms, is associated with a distinct clinical course. In such cases, clinicians are advised to integrate sustained supportive periodontal therapy, including biofilm-sustained supportive periodontal therapy, the application of topical antimicrobials, and biofilm suppression, with careful consideration of drug interactions. They should also address xerostomia or salivary dysfunction [34]. Advancements in the field of microbial diagnostics have led to the development of novel methods for the integration of sequencing and targeted quantitative polymerase chain reaction (qPCR) with host response biomarkers. These biomarkers, which include salivary matrix metalloproteinase-8 (MMP-8), have the potential to further enhance age-tailored monitoring and therapeutic decision-making [35].

### 3.3. Mechanistic Links and Clinical Implications Throughout the Lifespan

In obese individuals, age modifies the host context in which obesity-associated dysbiosis unfolds. In younger adults, obesity-related metaflammation may heighten their inflammatory response to a microbial challenge, amplify tissue-destructive signaling, and support rapid ecological turnover within periodontal biofilms [6,7,24,36]. In older adults, immunosenescence and inflammaging may reduce microbial clearance and resolution efficiency, while salivary dysfunction, multimorbidity, and care dependency alter the available niches and the ability to control plaque [17,18,19,20,29,30,37].

While biologically plausible, these proposed mechanisms and age-aware clinical reasoning are largely inferred from cross-sectional data, which cannot establish causality or track dynamic changes over time [38,39]. Therefore, they do not yet validate age-specific treatment algorithms or definitively prove distinct therapeutic pathways. The current evidence is better interpreted as suggesting different management priorities rather than proving distinct therapeutic pathways.

In younger obese adults, more overt inflammatory activity may justify early intensive biofilm control, careful metabolic risk assessment, and close monitoring of disease dynamics. Among older adults, supportive care, xerostomia management, maintenance adherence, and review of multimorbidity and polypharmacy may be especially important [40,41].

Biological age may be more informative than chronological age alone. Frailty, tooth retention, functional status, medication burden, and salivary reserve likely mediate much of the observed variability in microbiota composition and clinical presentation [42,43,44].

At the same time, obesity-related systemic inflammation, altered adipokine signaling, diet, smoking, and glycemic dysregulation remain major confounders that can modify periodontal dysbiosis across all age groups [38,45,46].

Therefore, the proposed age-aware trajectories should be considered a working model for hypothesis generation and study design, not a definitive classification.

The limitations of this review should be acknowledged. The literature is heterogeneous with respect to periodontal case definitions, obesity phenotyping, sampling sites, sequencing methods, and confounder adjustment. This variability severely limits the generalizability of findings and the ability to draw robust comparative conclusions between younger and older obese adults with periodontitis, underscoring the critical need for methodological standardization in future studies. Socioeconomic context, access to care, non-response, and representativeness are also poorly reported in many studies [47,48,49].

## 4. Discussion

### 4.1. Interpretation of the Evidence Base

This systematic review highlights a central constraint: although 50 adult studies were retained for qualitative synthesis, few adult human studies directly combined obesity or overweight, periodontal phenotyping, and oral microbiota profiling, and robust age-stratified analyses are rare. The most defensible conclusion is therefore that obesity is associated with oral microbial differences compatible with periodontal dysbiosis, while aging-related host and exposure factors are likely to modify these patterns rather than define proven age-specific signatures.

The plausibility of age as an effect modifier is supported by established concepts in immunometabolism and inflammaging. Metaflammation in obesity can alter leukocyte function and inflammatory signaling, whereas aging is associated with immune remodeling and chronic inflammatory tone [20,21,50,51,52]. In a dysbiosis-driven disease such as periodontitis [8,9,10], such host shifts can change selective pressures in the subgingival ecosystem and potentially influence which microbial strategies (inflammation exploitation, stress tolerance) dominate [53,54,55,56].

Importantly, oral microbiome studies are sensitive to local ecological sampling sites, periodontal case definition, and analytic pipeline choices [5,57,58]. Therefore, inconsistencies in reported taxa across studies do not necessarily refute an obesity–dysbiosis signal. Instead, they underscore the need for standardized phenotyping and harmonized microbiome workflows.

### 4.2. Clinical Implications

Although age-stratified microbial signatures in obese periodontitis patients remain insufficiently established, several practical implications emerge. In periodontal assessments, researchers should integrate systemic and behavioral factors that cluster with obesity (glycemic dysregulation, diet quality, and smoking) and aging (polypharmacy, xerostomia, functional limitation, and care dependency) [59,60,61].

Non-surgical periodontal therapy, supportive care, and interdisciplinary management of metabolic risk remain the best-supported clinical priorities. Evidence for differences in treatment response by age is suggestive rather than conclusive [19,62,63].

Antimicrobial stewardship is essential. Systemic antimicrobials may be considered in selected severe cases as adjuncts to mechanical therapy, but benefits must be weighed carefully against adverse effects, resistance, and drug-interaction risks, especially for older adults with multimorbidity [64,65].

Finally, salivary or crevicular biomarkers (e.g., MMP-8) and emerging point-of-care microbial panels may support monitoring of high-risk patients, but their incremental value beyond clinical staging/grading requires validation in age-stratified obesity cohorts [16,66,67,68].

### 4.3. Limitations

This review has several limitations. The evidence base is heterogeneous and largely observational, limiting causal inference. Key confounders, including smoking, diabetes control, diet, oral hygiene, socioeconomic status, access to care, medication burden, and tooth retention, were variably measured and adjusted. Many studies relied on clinic-based or convenience samples with limited information on non-response or representativeness.

The two-tier evidence approach was used to prevent overstatement: Tier 2 findings on age-linked exposure should be interpreted as modifiers that contextualize, rather than replace, the limited direct evidence from obese periodontitis cohorts. Therefore, conclusions about distinct age-specific microbial trajectories remain hypothesis-generating.

### 4.4. Future Directions

Future directions should emphasize the critical need for future studies to implement more robust and standardized sampling strategies to improve representativeness and reproducibility. This includes using prospective cohort designs with diverse populations to capture variability across age, sex, ethnicity, and socioeconomic status. Comprehensive adjustment for confounders such as smoking, diabetes control, diet, oral hygiene, medication burden, multimorbidity, and access to care must be prioritized to reduce bias and clarify causal relationships [69,70,71,72].

Mechanistic work integrating metagenomics/metatranscriptomics with host-response profiling (cytokines, neutrophil function, and barrier integrity) could clarify whether aging shifts periodontal dysbiosis toward persistence-oriented communities. Candidate taxa associated with stress tolerance and chronic inflammation (e.g., *Filifactor alocis*) merit particular attention as potential markers of biofilm resilience [73,74,75].

At the population level, addressing barriers to oral healthcare access among older adults with obesity remains essential, particularly in cases where mobility limitations, caregiving needs, and socioeconomic vulnerability reduce maintenance adherence [76,77,78].

## 5. Conclusions

This systematic review suggests that age likely modifies oral microbiota patterns associated with obesity and periodontitis, but the supporting evidence remains indirect, heterogeneous, and largely cross-sectional. Younger adults are more often aligned with a higher-activity, pathogen-rich inflammatory pattern, whereas older adults are more often aligned with lower-diversity, persistence-oriented communities. However, these patterns should be interpreted as working models rather than established subtypes.

Chronological age alone is an incomplete surrogate for vulnerability. Biological age markers such as frailty, multimorbidity, polypharmacy, tooth retention, and salivary hypofunction may better capture the host context in which dysbiosis develops and is expressed clinically.

Accordingly, the current evidence supports cautious age-aware interpretation and individualized periodontal care, but not definitive age-specific treatment algorithms. Given the indirect, heterogeneous, and largely cross-sectional nature of current evidence, robust longitudinal and interventional studies are critically needed to address these limitations and establish definitive age-specific microbiome-based recommendations.

## Figures and Tables

**Table 1:** **Representative human studies** contributing most directly to the age-aware synthesis of obesity, periodontitis, and the oral microbiota.

Study	Population/Age	Sample/Method	Main Contribution to the Age-Aware Synthesis
**Tam et al. 2018** [23] **Direct evidence**	18 adults with type 2 diabetes mellitus and mostly moderate/severe periodontitis; mean age 68.2 ± 6.8 years; obese versus non-obese	Deepest-site subgingival plaque plus unstimulated saliva; 16S rRNA sequencing (Illumina MiSeq)	Obesity was associated with altered community structure, including higher levels of *Fusobacterium, Prevotella*, and *Campylobacter* and lower levels of *Rothia and Haemophilus.*
**Rahman et al. 2023** [26] **Direct evidence**	75 adults aged 18–60; healthy-weight, overweight, and obese groups; mild versus moderate/severe periodontitis	Subgingival plaque; 16S rRNA sequencing (Nanopore)	Overweight or obesity with moderate/severe periodontitis was associated with enrichment of *Porphyromonas gingivalis, Tannerella forsythia, Prevotella intermedia*, and bridging taxa.
**Lé et al. 2023** [25] **Direct evidence**	45 adults with periodontitis; normal-weight versus obese; mean ages: 47.6 and 53.6 years	Subgingival plaque and saliva; 16S rRNA sequencing (Illumina)	Obese patients showed higher periodontal inflamed surface area, lower diversity, and a shifted microbial signature with reduced post-treatment stability.
**Maciel et al. 2016** [22] **Direct evidence**	166 adults with periodontal health or chronic periodontitis; obese versus normal-weight	Six-site subgingival biofilm; checkerboard DNA–DNA hybridization	Obesity was associated with higher levels and proportions of several periodontal pathogens, especially in chronic periodontitis.
**Thomas et al. 2021** [27] **Direct evidence**	19 adults with periodontitis; body mass index: 20–25 kg/m^2^ versus > 30 kg/m^2^	Saliva; microbiota sequencing and diversity analysis	Obesity was linked to poorer oral health, altered alpha diversity, and increased *Capnocytophaga* abundance; sex-related differences were also observed.
**de Andrade et al. 2021** [28] **Direct evidence**	77 young adults without destructive periodontal disease; normal-weight, overweight, and obese groups	Subgingival biofilm; checkerboard analysis	Overweight and obesity were associated with higher levels of Porphyromonas gingivalis and *Tannerella forsythia* before clearly destructive disease became evident.
**Genco et al. 2019** [24] **Contextual age study**	1206 older women aged 53–81 years with varying periodontal severity	Subgingival plaque; 16S rRNA gene sequencing (Illumina MiSeq)	Older-adult periodontal disease was associated with higher richness and enrichment of taxa such as *Tannerella forsythia, Porphyromonas gingivalis, Dialister*, and Filifactor alocis.
**Lira-Junior et al. 2018** [30] **Contextual age study**	441 adults spanning midlife and older age	Stimulated saliva; checkerboard DNA–DNA hybridization	Older participants showed higher counts of several periodontitis-associated taxa, including *Porphyromonas gingivalis, Tannerella forsythia,* and *Eubacterium nodatum*.
**Schwartz et al. 2021** [29] **Contextual age study**	Adult clinic cohort across young, middle-aged, and old-age groups	Saliva; 16S rRNA sequencing with multivariable analysis	Age-related compositional differences persisted after adjustment and were partly influenced by edentulism, treatment history, and polypharmacy.
**Eriksson et al. 2019** [31] **Contextual study**	40 adults with rheumatoid arthritis; mean age: 60.0 ± 11.0 years	Subgingival plaque and saliva; 16S rRNA sequencing	Moderate/severe periodontitis was associated with a periopathogen-rich profile and illustrates the confounding influence of systemic multimorbidity in older adults.

## Data Availability

The original contributions presented in the study are included in the article, further inquiries can be directed to the corresponding author.

## Supplementary Materials

The following supporting information can be downloaded at: https://www.mdpi.com/article/10.3390/nu18081256/s1, Table S1. NOS-informed quality appraisal of adult studies highlighted in the main synthesis; Table S2. Evidence map of the 50-publication synthesis corpus.
