# Supplementary material for: Age-Related Differences in Oral Microbiota Among Obese Patients with Periodontitis: A Systematic Review
Source: Nutrients. 2026 Apr 16;18(8):1256. doi: 10.3390/nu18081256 (PMC13119476; doi:10.3390/nu18081256)

Supplementary Table S1. NOS-informed quality appraisal of adult studies highlighted in the main synthesis

This supplementary table summarizes the qualitative interpretation of selection, comparability, and outcome/exposure domains for the ten adult studies highlighted in the revised manuscript. The judgments are intended to support interpretation of evidence rather than to function as exclusion criteria.

Study	Design / context	Overall concern	Main rationale
Tam et al. 2018	Case-control / clinic-based	Moderate-high concern	Small clinical sample; obese vs non-obese comparison was informative, but selection bias and limited comparability reduce certainty.
Rahman et al. 2023	Cross-sectional	High concern	Convenience sampling and limited confounder adjustment; useful sequencing data but age-aware inference remains indirect.
Lé et al. 2023	Prospective / interventional context	Moderate-high concern	Rich microbiome phenotyping, but modest sample size and obesity groups were not designed for direct age-stratified inference.
Maciel et al. 2016	Cross-sectional	Moderate concern	Larger adult sample and periodontal phenotype comparison strengthen inference, although residual confounding remains likely.
Thomas et al. 2021	Pilot study	High concern	Very small sample, limited generalizability, and sex-specific cohort design.
de Andrade et al. 2021	Cross-sectional	Moderate concern	Useful pre-disease signal in young adults,

			but not a destructive periodontitis cohort.
Genco et al. 2019	Observational cohort / older women	Moderate concern	Large, well-characterized older-adult cohort, though obesity was not the primary exposure of interest.
Lira-Junior et al. 2018	Observational	Moderate concern	Age-related oral microbial trends were informative, but the study was not designed around obesity-periodontitis-age interaction.
Schwartz et al. 2021	Observational	Moderate concern	Multivariable analysis improved interpretation, but saliva-based age effects were influenced by edentulism and treatment history.
Eriksson et al. 2019	Contextual comorbidity study	Moderate-high concern	Helpful for multimorbidity context, but rheumatoid arthritis introduces confounding for direct age-aware synthesis.

Supplementary Table S2. Evidence map of the 50-publication synthesis corpus

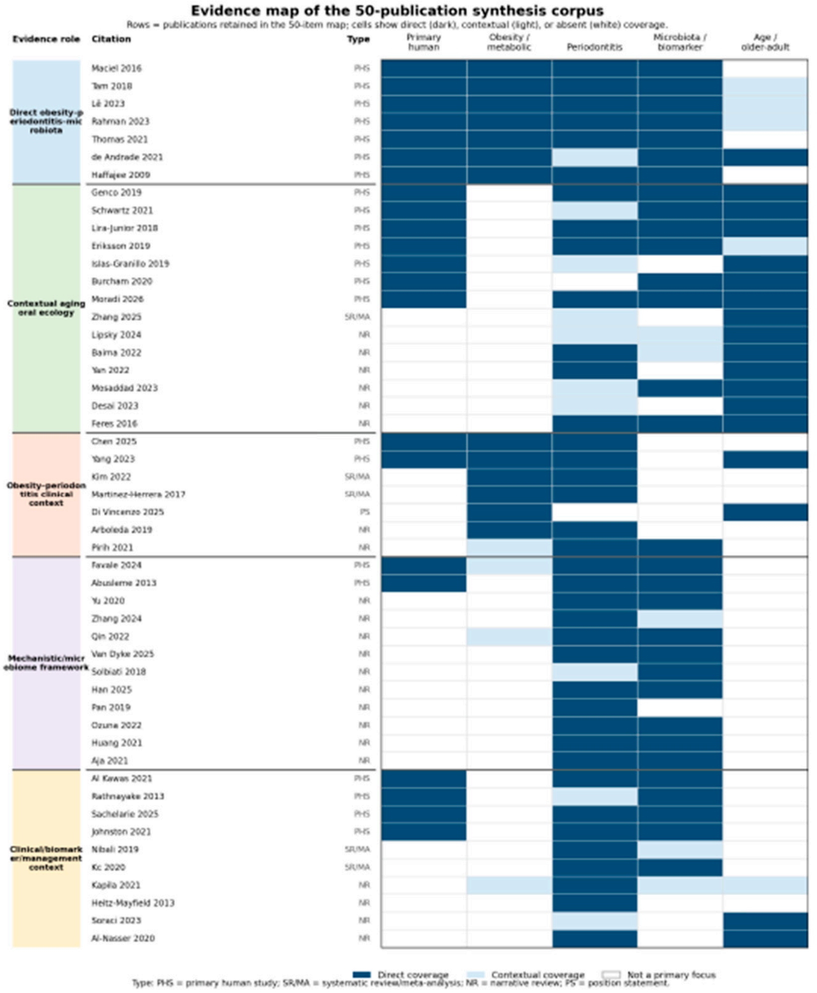

Supplement: Supplementary file 1 [file nutrients-18-01256-s001.zip › nutrients-4174456-supplementary.pdf]
